# Chromosome-level genome assembly and sex chromosome identification of the pink stem borer, *Sesamia inferens* (Lepidoptera: Noctuidae)

**DOI:** 10.1038/s41597-024-03625-1

**Published:** 2024-07-22

**Authors:** Xiao-Rui Yu, Xu Chen, Qing-Rong Bai, Ming-Yue Mu, Liang-De Tang, Guy Smagghe, Lian-Sheng Zang

**Affiliations:** 1 State Key Laboratory of Green Pesticide, Key Laboratory of Green Pesticide and Agricultural Bioengineering, Ministry of Education, Center for R&D of Fine Chemicals of Guizhou University, Guiyang, 550025 China; 2 Kweichow Moutai Group, Zunyi, 564501 China; 3 Institute of Entomology, Guizhou University, Guiyang, 550025 China

**Keywords:** Genomics, Evolutionary biology

## Abstract

The pink stem borer, *Sesamia inferens* Walker (Lepidoptera: Noctuidae), is one of the most notorious pest insects of rice and maize crops in the world. Here, we generated a high-quality chromosome-level genome assembly of *S. inferens*, using a combination of Illumina, PacBio HiFi and Hi-C technologies. The total assembly size was 973.18 Mb with a contig N50 of 33.39 Mb, anchored to 31 chromosomes, revealing a karyotype of 30 + Z. The BUSCO analysis indicated a high completeness of 98.90% (n = 5286), including 5172 (97.8%) single-copy BUSCOs and 58 (1.1%) duplicated BUSCOs. The genome contains 58.59% (564.58 Mb) repeat elements and 26628 predicted protein-coding genes. The chromosome-level genome assembly of *S. inferens* provides in-depth knowledge and will be a helpful resource for the Lepidoptera and pest control research communities.

## Background & Summary

Lepidoptera, encompassing butterflies and moths, is the second most diverse pest insect, with 180,000 described species. They commonly possess 31 chromosomes and constitute one-tenth of Earth’s described species^1^. Moreover, in both nature and agriculture settings, there is hardly any plant or crop that is not attacked by at least one lepidopteran pest^2–4^. Indeed, the larval stages (caterpillars) are major pests in forests, stored grains, and fiber and food crops. Besides, resistance to insecticide is an increasing problem and moths are among the most feared invasive species.

In the family of moths or Noctuidae, stem borers are notorious pest insects; the stem borer caterpillars damage crops by boring or tunneling inside their plant stems. The pink stem borer or *Sesamia inferens* Walker (Lepidoptera: Noctuidae) is very destructive for rice in the world^5–7^, but this polyphagous pest is also a major pest to a broad spectrum of crops, encompassing economically important graminaceous crops such as maize, sorghum, wheat, oats and sugarcane^8–10^. The adults can fly long distance, and the females release sex pheromone to attract the male for copulation, where the sex pheromones and the pheromone binding protein (PBP) gene family are relatively conserved in the Noctuidae and act according a lock-and-key principle^11–14^. After eclosion, adult moths engage in courtship and mating behavior in 0-day-old, with a mating rate reaching as high as 83.3%^15^; one female moth can produce 300–600 eggs. Hence, the females maximize their fitness by laying eggs preferentially on plants that maximize their offspring performance^16–21^. Our experimental results^22^, employing the age-stage, two-sex life table theory, and based on indoor experiments, along with statistical analyses of the offspring from oviposition and hatching of *S. inferens*, as well as other multiple parameters, have revealed its potential for widespread damage. Larvae tunnel through stalk internodes, weakening them and making them susceptible to breakage by strong winds, while also exposing plants to infection by the red rot fungus, leading to a significant decrease in sucrose content^23^. *S. inferens* successfully accomplished its entire developmental cycle on different gramineous crop hosts^22^ Symptoms known as “dead hearts” or “white heads”^24^, cause plant lodging and unfilled grains, leading to high yield losses^25–27^. Due to high levels of insecticide resistance and the hidden behavior of the insects into the plant stems, reducing the efficacy of chemical and biological control with parasitoids, the best options today for pest population suppression include field trapping using sex pheromones^22,28^ and the cultivation of trap plants^29,30^.

In this study, we present the first chromosome-level genome assembly and sex chromosome identification of *S. inferens*, providing valuable genomic resources for further research and development. The resulting assembly has a high quality, with a scaffold N50 size of 33.39 Mb. The completeness of the assembly was assessed using the BUSCO analysis, which revealed a high completeness of 98.90%. Repetitive elements were found to constitute a significant portion of the *S. inferens* genome, accounting for 58.59% of the total genome size. A total of 26628 protein-coding genes were identified in genome assembly. In conclusion, this chromosome-level assembly of the *S. inferens* genome does not only provide valuable genomic resources for understanding the biology and genetic basis for Lepidoptera, and supports the development of effective strategies for pest insect control based on sex pheromone traps and without use of chemical pesticides.

## Methods

### Sample collection and sequencing

#### Insect materials

**Fig. 1:**
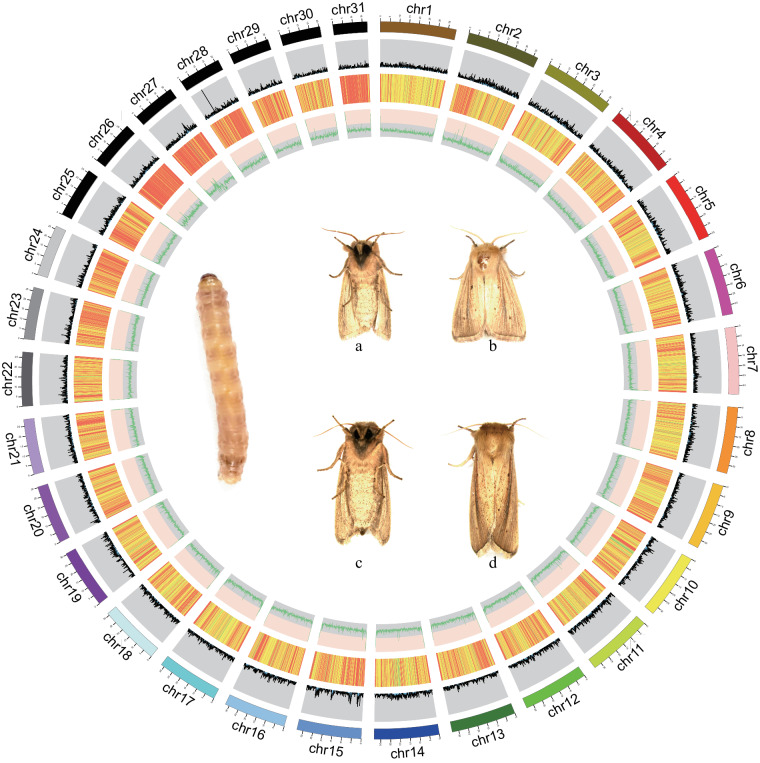
Chromosome-level genome assembly results information circle plot. From outer to inner layers: chromosome information; gene density; repeat sequence content; GC content; photograph of *Sesamia inferens* larva, male adult **(a, b)** and female adult **(c, d)**.

Specimens of *S. inferens* were collected from the Shibanzhen, Bozhou District, Zunyi City, Guizhou Province, China. The larvae were collected from sorghum plants (*Sorghum bicolor*) (Fig. 1). The samples included 3rd instar larvae, pupae, and adult males and females. Among them, 3rd instar larvae were subjected to 24-hour starvation treatment. To ensure the thorough removal of microbial contaminants from the surface of the samples, both larvae and pupae were subjected to surface sterilization. This process involved immersion in 75% ethanol for 1 min followed by three subsequent rinses with sterile water. The detailed protocols are as follows.

We extracted genomic DNA from *S. inferens* 3rd instar larvae samples using the Genome DNA extraction Kit (TIANGEN) as per the product manual. After extraction, utilizing the NanoDrop One, we detected the purity, concentration, and nucleic acid absorption peaks of *S. inferens* genomic DNA, focusing on the OD260/280 and OD260/230 ratios. For precise concentration determination, we employed the Qubit 3.0 system. A comparative analysis between Qubit 3.0 fluorescence photometer and NanoDrop One was conducted to assess sample purity. Additionally, we performed agarose gel electrophoresis to ascertain the integrity of the genomic DNA. For sequencing preparations, we employed Qubit 3.0 for precise quantification and Agilent 2100 Bioanalyzer for size analysis to ensure the library’s compliance with anticipated dimensions. Upon successful library validation, we initiated sequencing on the PacBio Sequel II (Pacific Biosciences), aligning the sequencing output with the predefined target data volume. The processed genomic DNA was subsequently employed for the generation of a single-molecule real-time (SMRT) bell sequencing library, utilizing the SMRTbell Template Prep Kit 2.0 developed by Pacific Biosciences^31^. As a result, we obtained a total of 80.0 Gb Illumina short read sequencing and 504.2 Gb PacBio sequencing reads. In total, 64.70 million raw reads (approximately 97.05 Gb) were obtained for scaffolding in genome assembly.

### Genome assembly

To achieve a high-quality assembly, we initiated the process with rigorous quality control of the initial raw reads. In the process of data quality control, several steps are implemented to ensure the integrity and reliability of the sequencing data. Initially, reads containing adapter sequences are eliminated. Following this, bases with consecutive quality scores below 20 at both ends of the sequencing read are subjected to trimming. Reads with a resulting length of less than 50 bp are subsequently excluded. Ultimately, only paired-end reads are retained for subsequent analysis. We used HiFiasm (v 0.15.1) to preliminarily assemble the *S. inferens* genome, which could resolve near-identical repeats and segmental duplications to generate better haplotype assemblies^32^. The HiFiasm outputs a primary assembly after performing all-versus-all read overlap alignment and correcting sequencing errors. Purge_Haplotigs software was used to complete genome deredundancy after initial assembly and error corrected, and the redundant heterozygous contigs were identified and removed according to reads depth distribution and sequence similarity^33^. The total length of HiFiasm, HiFiasm + purge haplotigs and HiFiasm + purge haplotigs + contamination removal was 99712 Mb, 97610 Mb and 97320 Mb, respectively (Table S1). The hybrid was used to obtain a *de novo* genome assembly for *S. inferens* with total length of 973.20 Mb and contig N50 length of 30.57 Mb (Table S2). The genome assembly quality was comprehensively evaluated through BUSCO alignment against the Lepidoptera_odb10 orthologue database, assessing the overall integrity of the assembled genome. After aligning the second-generation reads to the genome, mutations were identified using software samtools, picard and GATK. Homozygosity and heterozygosity rates for SNPs and InDels were then calculated separately. The homozygous SNP rate was found to be <0.001%, while the homozygous InDel rate was 0.001%. In contrast, the heterozygous SNP rate was 1.070%, and the heterozygous InDel rate was 0.247%.

### Chromosomal-level genome scaffolding with Hi-C data

To obtain the genome at the chromosomal level, Hi-C technology (High-throughput/resolution chromosome conformation capture) was applied^34,35^. The Hi-C library was prepared using a modified method according to standard protocol^36^. The samples were 3rd instar larvae.

**Fig. 2:**
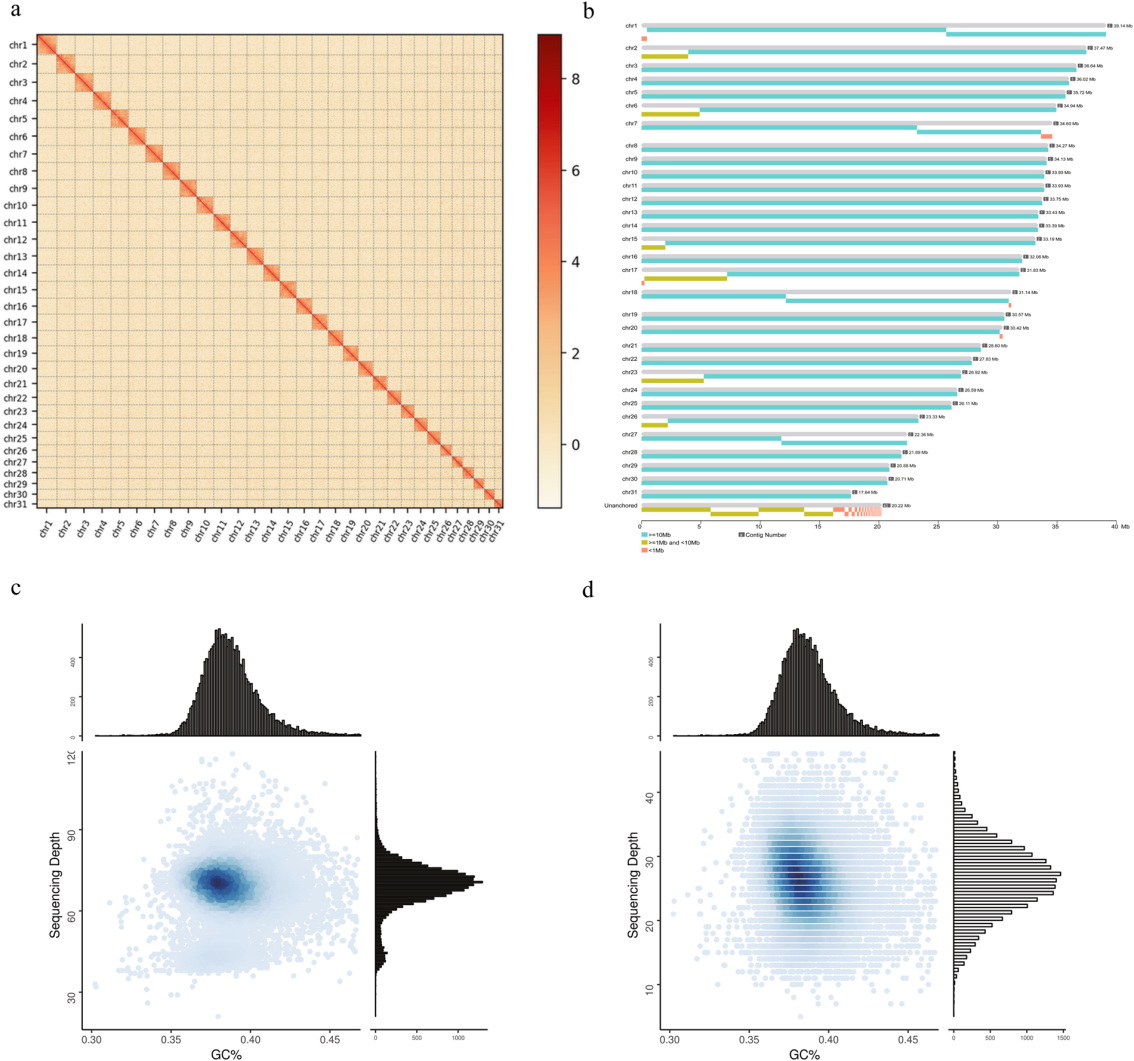
Genome assembly of *Sesamia inferens*. **(a)** Hi-C assembly of chromosome interactive heat map. Abscissa and ordinate represent order of each bin on corresponding chromosome group. Color block illuminates intensity of interaction from white (low) to red (high). **(b)** Contig distribution on genome chromosomes. The grey color represents the length of the corresponding chromosome, while other colors represent contigs of different length ranges. **(c)** Association analysis of GC content and coverage depth of second-generation reads. **(d)** Association analysis of GC content and coverage depth of third-generation reads.

Cells were treated with paraformaldehyde to fixed DNA conformation for 10 mins and stopped crosslinking by 2.5 M glycine for 20 mins. Subsequent to cell lysis, Crosslink DNAs were cut with a restriction enzyme and produced fill ends with biotin, DNA fragments were ligated using DNA ligase. To reverse the cross-linked state of DNA, proteinase digestion was applied, followed by purification of DNA, which was subsequently randomly sheared into fragments ranging from 300–500 bp. Biotin-labeled DNA was selectively captured using streptavidin magnetic beads, which was used to build the library and subsequent sequencing via the Illumina platform. We used bowtie 2 (v 2.2.3)^37^ to map the paired-end reads to the preliminary assembly. Then, HiC-Pro (v 2.7.8)^38^ was used to detect the ligation site of unmapped reads, which were mainly composed of the chimeric regions spanning across the ligation junction. High-quality clean data 94.998 Gb (read length: 150 bp) were generated after sequencing and filtering, then used for preliminary assembly by applying a 3D-DNA pipeline^35^ and LACHESIS^39^ using default parameters. We employed Juicer to construct the chromosome interaction map and then utilized Juicebox for visual correction. This allowed us to identify potential errors in contig sequence, direction, or assembly within the contig, ensuring the accuracy and reliability of our genome assembly. After the assisted assembly of the genome, a comprehensive genome-wide interaction map was constructed using Juicer^40^. Analysis of Hi-C data revealed assembly errors in the 3D-DNA assembly process, encompassing contig order, orientation, and internal arrangement. Performed manual visual error correction using JuiceBox (v 2.13.07)^40,41^. The corrected genome-wide interaction map exhibits enhanced intra-chromosomal interactions, with stronger interactions occurring between contigs in closer linear proximity. A chromatin contact matrix was manually curated in JuiceBox and the 31 scaffolds are clearly distinguishable in the heatmap in Fig. 2a, the interaction signal around the diagonal is strongly apparent. Contig distribution on genome chromosomes in Fig. 2b. The 88 contigs were divided, anchored, sorted, oriented, and merged into 31 chromosomes using LACHESIS and corrected by JuiceBox. The chromosomal heatmap showed good collinearity on the diagonal, which confirms the high quality of scaffolding. The final genome assembly was 973.18 Mb with a scaffold N50 of 33.39 Mb (Table 1, Fig. 1).

**Table 1:** Hi-C assisted assembly statistics for *Sesamia inferens*.

-genome version	Before Hi-C	After Hi-C		
	The whole genome	The whole genome	Chromosome	Free sequence
Sequence length (bp)	973,197,345	963,664,327	943,451,933	20,212,394
Sequence number	88	74	31	43
Contig N50 (bp)	30,574,287	30,166,720	30,574,287	3,840,887
Scaffold N50 (bp)	30,574,287	33,394,412	33,394,412	3,840,887

### Sex chromosomes identification

**Fig. 3:**
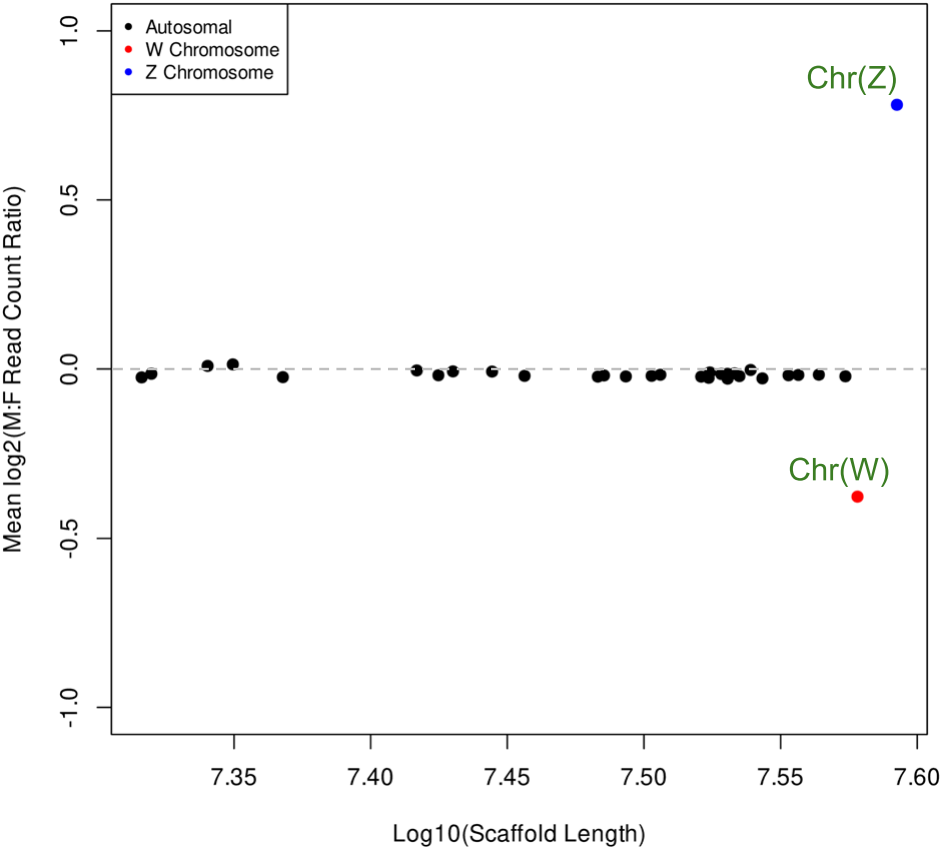
Identification of sex chromosomes in *Sesamia inferens* genome assembly. 10 male and 10 female adults were resequenced, and the obtained reads were analyzed for coverage comparison. Chromosomes with a log2 (M:F read counts) value of 0 were regarded as autosomes (black dots), that with a value less than or equal to the −0.25 were considered W chromosome (red dot), and that with a value greater than or equal to 0.25 were considered Z chromosome (blue dot).

In this study, we performed whole-genome resequencing of 10 male and 10 female adult of *S. inferens* using the Illumina platform and producing a total of 294.63 Gb clean data. Quality-controlled sequencing reads were aligned to the reference genome scaffolds using BWA software (v 0.7.17)^42^. The resulting BAM files were utilized for further coverage analysis. Coverage rates for males and females were calculated separately using Samtools (v 1.10)^43^. The inherent copy number differences between the sexes for sex chromosomes, where the Z chromosome exhibits a higher copy number in males, while the W chromosome is present only in females, were analyzed^44–46^. The log ratio of male to female coverage (log2(M:F)) was computed, and changepoint analysis was performed using the R package “changepoint” (https://CRAN.R-project.org/package=changepoint) to detect points of variation. Chromosomes were categorized based on their log2(M:F) values: chromosomes with values ranging from 0 to ±0.1 were considered autosomes; those with values less than −0.25 were designated as W chromosomes; and those with values greater than or equal to 0.25 were identified as Z chromosomes (ZZ: ♂; ZW: ♀). Based on the log2(M:F) ratio, chromosome 1 was identified as the Z chromosome, and chromosome 31 as the W chromosome (Fig. 3).

### Transcriptome sequencing

To assist in the annotation of genome structure, transcriptomic libraries were prepared from the 3rd instar larvae, pupae, adult males and adult females of *S. inferens*. Each sample designated for sequencing had an individual library constructed for the procedure. Total RNA was isolated from individual *S. inferens* sample utilizing the TRIzol (Invitrogen, Carlsbad, CA, USA) reagent method. Following homogenization, samples were allowed to stand at ambient conditions before chloroform was introduced. The mixture underwent centrifugation at 12,000 g at 4 °C, allowing for phase separation. The aqueous phase was subsequently subjected to isopropanol precipitation and centrifugation. The RNA pellet obtained was rinsed in 75% ethanol (prepared in RNase-free water) and centrifuged twice to ensure purity. The air-dried pellet was reconstituted in DEPC-treated water, and its integrity and concentration were quantified using a NanoDrop-2000 spectrophotometer at 260 nm. The RNA samples that had good quality were then utilized for cDNA library construction. Sequencing was carried out on the Illumina NovaSeq 6000 platform^47^. The obtained spliced transcript was used for genome structure annotation to provide evidence of transcription level.

### Genome quality assessment

The best five hits of BLASTN again NCBI NT database were from *Atethmia*, *Cosmia*, *Mythimna*, *Amphipyra* and *Xestia* (Table 2). Moreover, we compared the Lepidoptera_odb10 database using BUSCO. The assessment showed 98.9% of BUSCO genes were successfully detected, of which 98.9% were single copy and 1.1% duplicated (Table 3). The results of these evaluations indicate that the genome assembly has a high level of completeness and accuracy.

**Table 2:** Blast search of interrupted contig sequences in NCBI NT database.

Genus	Blast number	Genome split number	Hit number	Percentage of genome (%)	Percentage of hits (%)	Median identity (%)
Atethmia	17,543	97,369	97,246	18.02	18.04	85.94
Cosmia	15,796	97,369	97,246	16.22	16.24	86.49
Mythimna	10,326	97,369	97,246	10.61	10.62	86.41
Amphipyra	5,918	97,369	97,246	6.08	6.09	98.73
Xestia	5,223	97,369	97,246	5.36	5.37	83.8

**Table 3:** Statistical result of BUSCO evaluation results of genome assembly.

	Gene number	Percentage (%)
Complete BUSCOs (C)	5230	98.9
Complete and single-copy BUSCOs (S)	5172	97.8
Complete and duplicated BUSCOs (D)	58	1.1
Fragmented BUSCOs (F)	13	0.2
Missing BUSCOs (M)	43	0.9
Total BUSCO groups searched	5286	100

The assembled *S. inferens* genome size is 973.18 Mb with a scaffold N50 of 33.39 Mb (Fig. 1, Table S2), close to the estimated size in other Lepidoptera^48^. Using blobtools (v. 1.1.160), we created a blobplot to evaluate possible contamination of the contigs used for genome assembly (Fig. 2c,d). Taken together, these confidently confirm the accuracy of the chromosome scaffolding.

### Repeat sequence annotation

We identified repeat sequences and transposable elements (TEs) using the methods of *de novo* assembly^35^ and homologous prediction. First, we used RepeatModeler (v 2.0.2) (https://github.com/Dfam-consortium/RepeatModeler) to predict the repeat sequence with default parameters. Then, RepBase database^49^ and RepeatMasker (v 4.1.2) (https://github.com/rmhubley/RepeatMasker) were used to annotate the sequence homologs. The results showed that 564.58 Mb are repeat sequences, accounting for 58.59% of the *S. inferens* genome. Among these repeat sequences, most (24.51%) are long interspersed nuclear elements (LINEs), followed by 12.92% of unclassified elements, 10.75% of long terminal repeats (LTRs)^50–52^, 5.55% of short interspersed nuclear elements (SINEs), 5.38% of rolling-circles and only 4.82% of DNA elements (Table 4).

**Table 4:** Statistics of repetitive elements in the *Sesamia inferens* genome.

Repeat Classes	Number of Elements	Length (bp)	Percentage of genome (%)
Retroelements	1384664	393168508	40.80
1. SINEs	320015	53440801	5.55
2. LINEs	809664	236163430	24.51
3. LTR elements	254985	103564277	10.75
DNA transposons	121193	46411464	4.82
Rolling-circles	232060	51815948	5.38
Unclassified	752099	124515971	12.92
Total interspersed repeats	—	564578315	58.59
Small RNA	141743	26692427	2.77
Satellites	5	1370	0.00
Simple repeats	106464	4841345	0.50
Low complexity	15344	722271	0.07

### Gene prediction and function assignment

We annotated protein coding genes in the *S. inferens* genome using a pipeline that combines *de novo* prediction, homology searching and transcriptome evidence^48^. The repeat-masked genome was then subjected to further analysis according to the MAKER (v 3.01.03) genome annotation pipeline^53,54^. First, we utilized BRAKER (v 2) to construct the parametric species model for the *S. inferens* genome^55–57^. Next, we employed Trinity (v 2.14.0) to perform transcript splicing with the default parameters for genome structure annotation^58,59^. The obtained spliced transcript was used for genome structure annotation to provide evidence of transcription level. Finally, we executed MAKER incorporating the transcriptome, genome, parametric model of species, and the protein sequences of 10 other Lepidoptera (*Abrostola tripartita*, *Bombyx mori*, *Cnaphalocrocis medinalis*, *Habrosyne pyritoides*, *Helicoverpa armigera*, *Hyphantria cunea*, *Plutella xylostella*, *Spodoptera exigua*, *Spodoptera frugiperda* and *Spodoptera litura*) with good annotations down from InsectBase 2.0 (http://v2.insect-genome.com) as input data to predict genes^48^. A total of 26628 protein coding genes were annotated following the pipeline combined with above-mentioned three methods. Our comparative analysis between our genome assembly and the previously published chromosome-level assembly of *S. inferens*^60^ highlighted several key differences. Specifically, our genome assembly exhibited a larger genome size of 973.18 Mb compared to the previously published size of 865.04 Mb. Additionally, while the previous assembly consisted of 1135 contigs and 69 scaffolds, our assembly comprised 88 scaffolds. Notably, our assembly featured a higher Contig N50 value of 30.17 Mb and a slightly lower Scaffold N50 value of 33.39 Mb. Furthermore, our analysis included the identification of the sex chromosomes of *S. inferens*, providing further elucidation of its karyotype, as detailed in Table S3.

### Phylogeny

**Fig. 4:**
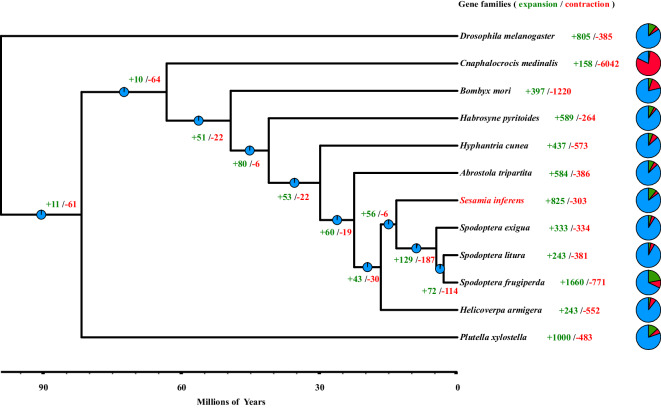
Phylogenetic analysis of *Sesamia inferens* and 10 other Lepidoptera species.

OrthoFinder^61–63^ (v 2.5.1) was used to analyze the orthologous and paralogous genes of 10 insect genomes, including *Drosophila melanogaster* (assembly accession: GCF_000001215.4), *P. xylostella* (assembly accession: GCA_019096205.1)*, A. tripartita* (assembly accession: GCA_905340225.1)*, B. mori* (assembly accession: GCF_014905235.1), *C. medinalis* (IBG_00192)*, H. pyritoides* (assembly accession: GCA_907165245.1), *H. cunea* (assembly accession: GCA_003709505.1), *S. frugiperda* (assembly accession: GCF_011064685.1), *S. exigua* (assembly accession: GCA_011316535.1), *S. litura* (assembly accession: GCA_002706865.1), *H. armigera* (assembly accession: GCF_002156985.1), and *D. melanogaster* was selected as an outgroup (Fig. 4).

Phylogenetic trees were constructed based on single-copy orthologous gene families. The phylogenetic tree was constructed by maximum likelihood (ML) using IQ-TREE (v 2.1.2) with the best model (JTT + F + R5) and 1000 rapid bootstrap replicates to assess the robustness of the tree^64^. Additionally, we used Astral-III^65^ to merge all gene trees obtained through OrthoFinder into a unified species tree. It is essential to emphasize that the two trees generated from these methods must be congruent, validating the consistency and accuracy of our phylogenetic analysis. Divergence time was estimated by MCMCtree^66^ program in the PAML package (v 4.8) based on the multiple sequence alignment protein sequences. The calibration time points of *D. melanogaster* (99.15 MYA), *P. xylostella* (81.66 MYA), *S. inferens* (13.26 MYA), *B. mori* (49.25 MYA) and *H. pyritoides* (40.98 MYA) were obtained from TimeTree^67^ (http://timetree.org/) (Fig. 4). Gene family contraction and expansion were analyzed using CAFE (v 4.2), incorporating the results from OrthoFinder and the phylogenetic tree with divergence time information^68^. Finally, iTOL (https://itol.embl.de/#) was used to visualize and enhance the appearance of the phylogenetic tree. *S. inferens* exhibits an explanation of 825, which is equivalent to half of that observed in *S. frugiperda*, lower than in *P. xylostella*, and on par with that in *D. melanogaster*. The expansion of gene families is considered a pivotal factor contributing to biodiversity and evolution. Data regarding gene family expansion in *S. inferens* reveals a relatively rapid rate of renewal and iteration. This accelerated gene family evolution enables the organism to adapt to the diverse and continually changing challenges presented by its environment. This result aligns with the phenomenon observed in the field, where the infection of *S. inferens* has transitioned gradually from localized edge infections to widespread field infestations^69^.

## Data Records

The raw sequencing data and genome assembly of *S. inferens* have been deposited at the National Center for Biotechnology Information (NCBI). Illumina, PacBio and Hi-C data for *S. inferens* genome sequencing have been deposited in the NCBI Sequence Read Archive with accession number SRR26501366, SRR27137600 and SRR27032946 under BioProject accession number PRJNA1014234^70^.

Illumina transcriptome data 3rd instar larva (SRR26056362), pupa (SRR26056882), female adult (SRR26050603), male adult (SRR26056479) are available under Bioproject PRJNA1014234^70^.

Genome resequencing data for female adults (SRR28744322, SRR28744323, SRR28744324, SRR28744325, SRR28744326, SRR28744327, SRR28744328, SRR28744328, SRR28744330, SRR28744331) and male adults (SRR28778051, SRR28778052, SRR28778053, SRR28778054, SRR28778055, SRR28778056, SRR28778057, SRR28778058, SRR28778059, SRR28778060) are available under Bioproject PRJNA1014234^70^.

This Whole Genome Shotgun project has been deposited at GenBank under the accession JAYKGN000000000^71^. The version described in this paper is version JAYKGN010000000.

The annotation file is available in *figshare*^72^.

## Technical Validation

After extraction, the DNA purity, concentration and integrity were detected using NanoDrop One, Qubit 3.0 fluorescence photometer and Agilent 2100 Bioanalyzer (Agilent Technologies, CA, USA), respectively. RNA integrity and concentration were quantified using a NanoDrop One spectrophotometer (Thermo Fisher Scientific, Waltham, MA, United States). High-quality DNA and RNA were used for sequencing.

We used three methods to assess the completeness and quality of the assembly. First, a data accuracy assessment was conducted to confirm the belonging of the assembly results to the target species. The genome sequence was fragmented at 10 kb intervals, and the resulting sequences were aligned to the NCBI nucleotide database (NT library) using Blast software^73^. Second, a sequence consistency evaluation was performed by aligning second and third-generation data to the assembled genome using BWA (v 0.7.17)^42^ and Minimap2 (v 2.24)^74^. As depicted in Table 5, The alignment statistics for the second-generation reads show a mapping rate of 99.67%, a paired mapping rate of 92.40%, an average sequencing depth of 69.38 X, and 99.98% coverage. For third-generation reads, the mapping rate was 99.98%, the average sequencing depth was 26.77 X, and the coverage was 100.00%. Higher mapping and coverage rates indicate a higher consistency between the assembly results and the reads, reflecting better assembly performance. Third, the quality of the genome sequence was evaluated by BUSCO (v 4)^75–78^ with Lepidoptera_odb10 and default parameters. In addition, after aligning second-generation reads to the genome, mutations were identified using samtools, picard, and GATK (v 4.4.0.0)^79^. The rates of homozygous and heterozygous SNPs and InDels were calculated. The homozygous SNP rate was <0.01%, the homozygous InDel rate was 0.001%, the heterozygous SNP rate was 1.070%, and the heterozygous InDel rate was 0.247%.

**Table 5:** Statistical results of reads alignment.

	Second-Generation Result	Third-Generation Result
Read mapping rate (%)	99.67	99.98
Paired mapping rate (%)	92.40	—
Genome average sequencing depth (×)	69.38	26.77
Coverage of genome (%)	99.98	100
Coverage of genome > 4 × (%)	99.95	99.94
Coverage of genome > 10 × (%)	99.85	97.73
Coverage of genome > 20 × (%)	99.28	72.83

### Supplementary information

supplementary Tables for main document

## Data Availability

No specific script was used in this work. All commands and pipelines used in data processing were executed according to the manual and protocols of the corresponding bioinformatic software, and default parameters were applied if not mentioned in the Methods described above.

## References

[1] Wright, C. J. *et al*. Comparative genomics reveals the dynamics of chromosome evolution in Lepidoptera. *Nat Ecol Evol.* **8**(4), 777–790 (2024). 38383850 10.1038/s41559-024-02329-4 PMC11009112

[2] Bilal, M. *et al*. Indoxacarb-loaded fluorescent mesoporous silica nanoparticles for effective control of *Plutella xylostella* L. with decreased detoxification enzymes activities. *Pest. Manag. Sci.* **76**, 3749–3758 (2020). 32431091 10.1002/ps.5924

[3] Oerke, E. Crop losses to pests. *J. Agr. Sci.* **144**, 31–43 (2005). 10.1017/S0021859605005708

[4] Mulhair, P. O. *et al*. Diversity, duplication, and genomic organization of homeobox genes in Lepidoptera. *Genome Res.* **33**, 32–44 (2023). 36617663 10.1101/gr.277118.122 PMC9977156

[5] Han, L. *et al*. Lethal and Sub - Lethal Effects of Transgenic Rice Containing *cry1Ac* and *CpTI* Genes on the Pink Stem Borer, *Sesamia inferens* (Walker). *Agr. Sci. China* **10**, 384–393 (2011). 10.1016/S1671-2927(11)60017-5

[6] Yang, L. *et al*. Floating chitosan-alginate microspheres loaded with chlorantraniliprole effectively control *Chilo suppressalis* (Walker) and *Sesamia inferens* (Walker) in rice fields. *Sci. Total. Environ.* **783**, 147088 (2021). 34088145 10.1016/j.scitotenv.2021.147088

[7] Soujanya, P. L. *et al*. Role of morphological traits and cell wall components in imparting resistance to pink stem borer, *Sesamia inferens* Walker in maize. *Front. Plant. Sci.* **14**, 1167248 (2023). 37554561 10.3389/fpls.2023.1167248 PMC10406494

[8] Chai, H. N. & Du, Y. Z. The complete mitochondrial genome of the pink stem borer, *Sesamia inferens*, in comparison with four other Noctuid moths. *Int. J. Mol. Sci.* **13**, 10236–10256 (2012). 22949858 10.3390/ijms130810236 PMC3431856

[9] Mahesh, P. *et al*. Natural Incidence of *Sesamia inferens* Walker, in Sugarcane Germplasm. *Sugar Tech.* **15**, 384–389 (2013). 10.1007/s12355-013-0212-2

[10] Cheraghali, Z. *et al*. Genetic diversity of populations of the stem borer *Sesamia nonagrioides* (Lepidoptera: Noctuidae) in southern and southwestern Iran, using RAPD-PCR. *North-West J. Zool.* **11**(1), 70–75 (2015).

[11] Wang, H. *et al*. Structural basis for action by diverse antidepressants on biogenic amine transporters. *Nature* **503**, 141–145 (2013). 24121440 10.1038/nature12648 PMC3904662

[12] Diéguez, M., Pàmies, O. & Moberg, C. Self-Adaptable Tropos Catalysts. *Accounts. Chem. Res.* **54**, 3252–3263 (2021). 10.1021/acs.accounts.1c00326 PMC8382228 34347444

[13] Jiang, N. *et al*. Revisiting the sex pheromone of the fall armyworm *Spodoptera frugiperda*, a new invasive pest in South China. *Insect Sci.* **29**, 865–878 (2021). 34297483 10.1111/1744-7917.12956

[14] Ando, T., Inomata, S. & Yamamoto, M. Lepidopteran sex pheromones. *Topics Curr. Chem.* **239**, 51–96 (2004). 10.1007/b95449 22160231

[15] Nagayama, A. *et al*. Emergence and mating behavior of the pink borer, *Sesamia inferens* (Walker) (Lepidoptera: Noctuidae). *Appl. Entomol. Zool.* **39**, 625–629 (2004). 10.1303/aez.2004.625

[16] Mayhew, P. J. Adaptive patterns of host-plant selection by phytophagous insects. *Oikos* **79**, 417–428 (1997). 10.2307/3546884

[17] Gripenberg, S., Mayhew, P. J., Parnell, M. & Roslin, T. A meta-analysis of preference-performance relationships in phytophagous insects. *Ecol. Lett.* **13**, 383–393 (2010). 20100245 10.1111/j.1461-0248.2009.01433.x

[18] Valladares, G. Host-Plant Selection in the Holly Leaf-Miner: Does Mother Know Best? *J. Anim. Ecol.* **60**, 227–240 (1991). 10.2307/5456

[19] Jaenike, J. On optimal oviposition behavior in phytophagous insects. *Theor. Popul. Biol.* **14**, 350–356 (1978). 751265 10.1016/0040-5809(78)90012-6

[20] Sekhar, J. C. *et al*. Differential Preference for Oviposition by *Sesamia inferens* Walker on Maize Genotypes. *Annals of Plant Protection*. *Sciences* **17**, 46–49 (2009).

[21] Zang, L., Wang, S., Zhang, F. & Desneux, N. Biological Control with *Trichogramma* in China: History, Present Status and Perspectives. *Annu. Rev. Entomol.* **66**, 463–484 (2020). 32976724 10.1146/annurev-ento-060120-091620

[22] Chen, L. *et al*. Demography and fitness of *Sesamia inferens* Walker (Lepidoptera: Noctuidae) on three important gramineous crops. *CABI Agric Biosci* **4**, 49 (2023). 10.1186/s43170-023-00191-1

[23] Nagayama, A. *et al*. Reinvestigation of sex pheromone components and attractiveness of synthetic sex pheromone of the pink borer, *Sesamia inferens* Walker (Lepidoptera: Noctuidae) in Okinawa. *Appl. Entomol. Zool.* **41**, 399–404 (2006). 10.1303/aez.2006.399

[24] Dey, A. *et al*. Molecular diversity of *Sesamia inferens* (Walker, 1856) (Lepidoptera: Noctuidae) from India. *3 Biotech.* **11**, 134 (2021). 33680699 10.1007/s13205-021-02678-y PMC7897588

[25] Jiang, M. X. & Cheng, J. A. Interactions between the striped stem borer *Chilo suppressalis* (Walk.) (Lep., Pyralidae) larvae and rice plants in response to nitrogen fertilization. *Anz. Schadl-j. Pest. Sc* **76**, 124–128 (2003). 10.1007/s10340-003-0001-x

[26] Rao, A. B. Technique of scoring for resistance in maize stalk borer (*S. inferens*.). In: Techniques for scoring for resistance to the major insect pests of maize. (AICMIP, IARI, New Delhi, 1983).

[27] Siddiqui, K. H. & Marwaha, K. K. The Vistas of Maize Entomology in India. (Kalyani Publishers, 1993).

[28] Wang, C. *et al*. Characterization of the pheromone receptors in Mythimna loreyi reveals the differentiation of sex pheromone recognition in Mythimna species. *Insect Sci.* **31**(1), 173–185 (2024). 37269179 10.1111/1744-7917.13215

[29] Gurr, G. M. *et al*. Multi-country evidence that crop diversification promotes ecological intensification of agriculture. *Nat. Plants* **2**, 16014 (2016). 27249349 10.1038/nplants.2016.14

[30] Tang, L. D. *et al*. Dead-end trap plants as an environment-friendly IPM tool: A case study of the successful use of vetiver grass in China. *Entomologia Generalis* **44**(1), 81–93 (2024). 10.1127/entomologia/2023/2194

[31] Eid, J. S. *et al*. Real-Time DNA Sequencing from Single Polymerase Molecules. *Science* **323**, 133–138 (2009). 19023044 10.1126/science.1162986

[32] Cheng, H. Y. *et al*. Haplotype-resolved de novo assembly using phased assembly graphs with hifiasm. *Nat. Methods* **18**, 170–175 (2021). 33526886 10.1038/s41592-020-01056-5 PMC7961889

[33] Roach, M. J., Schmidt, S. A. & Borneman, A. R. Purge Haplotigs: allelic contig reassignment for third-gen diploid genome assemblies. *BMC Bioinformatics* **19**, 460 (2018). 30497373 10.1186/s12859-018-2485-7 PMC6267036

[34] van Berkum, N. L. *et al*. Hi-C: A Method to Study the Three-dimensional Architecture of Genomes. *J. Vis. Exp.* **39**, 1869 (2010). 10.3791/1869 PMC3149993 20461051

[35] Dudchenko, O. *et al*. De novo assembly of the *Aedes aegypti* genome using Hi-C yields chromosome-length scaffolds. *Science* **356**, 92–95 (2017). 28336562 10.1126/science.aal3327 PMC5635820

[36] Wingett, S. *et al*. HiCUP: pipeline for mapping and processing Hi-C data. *F1000Res.* **4**, 1310 (2015). 26835000 10.12688/f1000research.7334.1 PMC4706059

[37] Langmead, B. & Salzberg, S. L. Fast gapped-read alignment with Bowtie 2. *Nat. Methods* **9**, 357–359 (2012). 22388286 10.1038/nmeth.1923 PMC3322381

[38] Kelly, S. T. & Yuhara, S. HiCUP-Plus: a fast open-source pipeline for accurately processing large scale Hi-C sequence data. *bioRxiv*. (2022).

[39] Burton, J. N. *et al*. Chromosome-scale scaffolding of de novo genome assemblies based on chromatin interactions. *Nat. Biotechnol.* **31**, 1119–1125 (2013). 24185095 10.1038/nbt.2727 PMC4117202

[40] Robinson, J. T. *et al*. Juicebox.js Provides a Cloud-Based Visualization System for Hi-C Data. *Cell Syst.* **6**, 256–258.e1 (2017). 10.1016/j.cels.2018.01.001 PMC6047755 29428417

[41] Durand, N. C. *et al*. Juicebox Provides a Visualization System for Hi-C Contact Maps with Unlimited Zoom. *Cell Syst.* **3**(1), 99–101 (2016). 27467250 10.1016/j.cels.2015.07.012 PMC5596920

[42] Li, H. Aligning sequence reads, clone sequences and assembly contigs with BWA-MEM. arXiv: Genomics (2013).

[43] Li, H. *et al*. The Sequence Alignment/Map format and SAMtools. *Bioinformatics.* **25**(16), 2078–2079 (2009). 19505943 10.1093/bioinformatics/btp352 PMC2723002

[44] Xu, H. *et al*. Chromosome-level genome assembly of an agricultural pest, the rice leaffolder Cnaphalocrocis exigua (Crambidae, Lepidoptera). *Mol. Ecol. Resour.* **22**(1), 307–318 (2022). 34228883 10.1111/1755-0998.13461

[45] Zhao, X. *et al*. A chromosome-level genome assembly of rice leaffolder, *Cnaphalocrocis medinalis*. *Mol. Ecol. Resour.* **21**(2), 561–572 (2021). 33051980 10.1111/1755-0998.13274

[46] Mongue, A. J. *et al*. Neosex chromosomes in the monarch butterfly, *Danaus plexippus. G3 (Bethesda).* **7**(10), 3281–3294 (2017). 28839116 10.1534/g3.117.300187 PMC5633379

[47] Rao, S. S. P. *et al*. A 3D Map of the Human Genome at Kilobase Resolution Reveals Principles of Chromatin Looping. *Cell* **159**, 1665–1680 (2014). 25497547 10.1016/j.cell.2014.11.021 PMC5635824

[48] Mei, Y. *et al*. InsectBase 2.0: a comprehensive gene resource for insects. *Nucleic. Acids. Res.* **50**, D1040–D1045 (2021). 10.1093/nar/gkab1090 PMC8728184 34792158

[49] Bao, W., Kojima, K. K. & Kohany, O. Repbase Update, a database of repetitive elements in eukaryotic genomes. *Mob. DNA* **6**, 11 (2015). 26045719 10.1186/s13100-015-0041-9 PMC4455052

[50] Xu, Z. & Wang, H. LTR_FINDER: an efficient tool for the prediction of full-length LTR retrotransposons. *Nucleic. Acids. Res.* **35**, W265–268 (2007). 17485477 10.1093/nar/gkm286 PMC1933203

[51] Ou, S. & Jiang, N. LTR_retriever: A Highly Accurate and Sensitive Program for Identification of Long Terminal Repeat Retrotransposons. *Plant Physiol.* **176**, 1410–1422 (2017). 29233850 10.1104/pp.17.01310 PMC5813529

[52] Ou, S., Chen, J. & Jiang, N. Assessing genome assembly quality using the LTR Assembly Index (LAI). *Nucleic. Acids. Res.* **46**, e126 (2018). 30107434 10.1093/nar/gky730 PMC6265445

[53] Cantarel, B. L. *et al*. MAKER: an easy-to-use annotation pipeline designed for emerging model organism genomes. *Genome Res.* **18**, 188–196 (2007). 18025269 10.1101/gr.6743907 PMC2134774

[54] Holt, C. & Yandell, M. MAKER2: an annotation pipeline and genome-database management tool for second-generation genome projects. *BMC Bioinformatics* **12**, 491 (2011). 22192575 10.1186/1471-2105-12-491 PMC3280279

[55] Brůna, T. *et al*. BRAKER2: automatic eukaryotic genome annotation with GeneMark-EP+ and AUGUSTUS supported by a protein database. *Nar. Genom. Bioinform.* **3**, lqaa108 (2020). 10.1093/nargab/lqaa108 PMC7787252 33575650

[56] Hoff, K. J. *et al*. Whole-Genome Annotation with BRAKER. *Methods Mol. Biol.* **1962**, 65–95 (2019). 31020555 10.1007/978-1-4939-9173-0_5 PMC6635606

[57] Hoff, K. J. *et al*. BRAKER1: Unsupervised RNA-Seq-Based Genome Annotation with GeneMark-ET and AUGUSTUS. *Bioinformatics* **32**, 767–769 (2015). 26559507 10.1093/bioinformatics/btv661 PMC6078167

[58] Grabherr, M. G. *et al*. Trinity: reconstructing a full-length transcriptome without a genome from RNA-Seq data. *Nat. Biotechnol.* **29**, 644–652 (2011). 21572440 10.1038/nbt.1883 PMC3571712

[59] Haas, B. J. *et al*. *De novo* transcript sequence reconstruction from RNA-seq using the Trinity platform for reference generation and analysis. *Nat. Protoc.* **8**, 1494–1512 (2013). 23845962 10.1038/nprot.2013.084 PMC3875132

[60] Li, H. R. *et al*. A chromosome-level genome assembly of *Sesamia inferens*. *Sci Data* **11**, 134, 10.1038/s41597-024-02937-6 (2024). 38272921 10.1038/s41597-024-02937-6 PMC10810861

[61] Emms, D. M. & Kelly, S. OrthoFinder: phylogenetic orthology inference for comparative genomics. *Genome Biol.* **20**, 238 (2019). 31727128 10.1186/s13059-019-1832-y PMC6857279

[62] Emms, D. M. & Kelly, S. OrthoFinder: solving fundamental biases in whole genome comparisons dramatically improves orthogroup inference accuracy. *Genome Biol.* **16**, 157 (2015). 26243257 10.1186/s13059-015-0721-2 PMC4531804

[63] Mier, P. & Pérez-Pulido, A. J. orthoFinder: a new automated tool for searching orthologous proteins useful for functional annotation. *F1000Res.* **5**, 1743 (2014).

[64] Nguyen, L. T. *et al*. IQ-TREE: A Fast and Effective Stochastic Algorithm for Estimating Maximum-Likelihood Phylogenies. *Mol. Biol. Evol.* **32**, 268–274 (2014). 25371430 10.1093/molbev/msu300 PMC4271533

[65] Zhang, C. *et al*. ASTRAL-III: polynomial time species tree reconstruction from partially resolved gene trees. *BMC Bioinformatics* **19**(Suppl 6), 153 (2018). 29745866 10.1186/s12859-018-2129-y PMC5998893

[66] Puttick, M. N. MCMCtreeR: functions to prepare MCMCtree analyses and visualize posterior ages on trees. *Bioinformatics* **35**, 5321–5322 (2019). 31292621 10.1093/bioinformatics/btz554

[67] Kumar, S. *et al*. TimeTree: A Resource for Timelines, Timetrees, and Divergence Times. *Mol. Biol. Evol.* **34**, 1812–1819 (2017). 28387841 10.1093/molbev/msx116

[68] Han, M. V. *et al*. Estimating gene gain and loss rates in the presence of error in genome assembly and annotation using CAFE 3. *Mol. Biol. Evol.* **30**, 1987–1997 (2013). 23709260 10.1093/molbev/mst100

[69] Sun, M. *et al*. Characterization and Expression of Genes Encoding Three Small Heat Shock Proteins in *Sesamia inferens* (Lepidoptera: Noctuidae). *Int. J. Mol. Sci.* **15**, 23196–23211 (2014). 25514417 10.3390/ijms151223196 PMC4284760

[70] *NCBI Sequence Read Archive* https://identifiers.org/ncbi/insdc.sra:SRP460199 (2023).

[71] Yu, X. R. *Sesamia inferens* isolate XY-2023, whole genome shotgun sequencing project. *GenBank* https://identifiers.org/ncbi/insdc:JAYKGN000000000 (2023).

[72] Yu, X. R. Chromosome-level genome assembly of Pink stem borer, *Sesamia inferens* Walker, 1856 (Lepidoptera: Noctuidae). *figshare*10.6084/m9.figshare.24418837.v1 (2023). 10.6084/m9.figshare.24418837.v1

[73] Johnson, M. *et al*. NCBI BLAST: a better web interface. *Nucleic. Acids. Res.* **36**, W5–9 (2008). 18440982 10.1093/nar/gkn201 PMC2447716

[74] Li, H. Minimap2: pairwise alignment for nucleotide sequences. *Bioinformatics* **34**, 3094–3100 (2017). 10.1093/bioinformatics/bty191 PMC6137996 29750242

[75] Simão, F. A. *et al*. BUSCO: assessing genome assembly and annotation completeness with single-copy orthologs. *Bioinformatics* **31**, 3210–3212 (2015). 26059717 10.1093/bioinformatics/btv351

[76] Manni, M. *et al*. BUSCO Update: Novel and Streamlined Workflows along with Broader and Deeper Phylogenetic Coverage for Scoring of Eukaryotic, Prokaryotic, and Viral Genomes. *Mol. Biol. Evol.* **38**, 4647–4654 (2021). 34320186 10.1093/molbev/msab199 PMC8476166

[77] Seppey, M., Manni, M. & Zdobnov, E. M. BUSCO: Assessing Genome Assembly and Annotation Completeness. *Methods Mol. Biol.* **1962**, 227–245 (2019). 31020564 10.1007/978-1-4939-9173-0_14

[78] Manni, M. *et al*. BUSCO: Assessing Genomic Data Quality and Beyond. *Curr. Protoc.* **1**, e323 (2021). 34936221 10.1002/cpz1.323

[79] McKenna, A. *et al*. The Genome Analysis Toolkit: a MapReduce framework for analyzing next-generation DNA sequencing data. *Genome Res.* **20**, 1297–1303 (2010). 20644199 10.1101/gr.107524.110 PMC2928508

