# Supplementary material for: Chromosome-level genome assembly and sex chromosome identification of the pink stem borer, Sesamia inferens (Lepidoptera: Noctuidae)
Source: Sci Data. 2024 Jul 22;11:810. doi: 10.1038/s41597-024-03625-1 (PMC11263549; doi:10.1038/s41597-024-03625-1)
Supplement: Supplementary file 1 — supplementary Tables for main document [file 41597_2024_3625_MOESM1_ESM.docx]

**Chromosome-level genome assembly and sex chromosome identification of the pink stem borer, *Sesamia inferens* (Lepidoptera: Noctuidae)**

Xiao-Rui Yu^1^, Xu Chen^1^, Qing-Rong Bai^1^, Ming-Yue Mu^2^, Liang-De Tang^1^, Guy Smagghe^3^, Lian-Sheng Zang^1^

**Affiliations**

1. State Key Laboratory of Green Pesticide, Key Laboratory of Green Pesticide and Agricultural Bioengineering, Ministry of Education, Center for R&D of Fine Chemicals of Guizhou University, Guiyang 550025, China

2. Kweichow Moutai Group, Zunyi 564501, China

3. Institute of Entomology, Guizhou University, Guiyang 550025, China

corresponding author(s): Lian-Sheng Zang (lsz0415@163.com), Qing-Rong Bai (bbbqqqrrr@163.com)

^#^These authors contributed equally to this work.

^*^To whom correspondence should be addressed: Lian-Sheng Zang (lsz0415@163.com), Qing-Rong Bai (bbbqqqrrr@163.com).

Table S1. Genome assembly results.

| **Mode** | **Total length (bp)** | **Total number** | **Total number (>= 2 kb)** | **max length (bp)** | **N50 (bp)** | **N90 (bp)** | **GC content (%)** |
| --- | --- | --- | --- | --- | --- | --- | --- |
| Hifiasm | 997,117,156 | 190 | 190 | 36,793,432 | 30,574,287 | 11,783,811 | 39.01 |
| hifiasm+purge_haplotigs | 976,104,368 | 129 | 129 | 36,793,432 | 30,574,287 | 12,151,824 | 38.88 |
| hifiasm+purge_haplotigs+contamination removal | 973,197,345 | 88 | 88 | 36,793,432 | 30,574,287 | 13,467,797 | 38.9 |

Table S2. Assembly statistics of *Sesamia inferens* genome.

|  | ***De novo*** | | ***Hi-C scaffolds*** | |
| --- | --- | --- | --- | --- |
|  | **Contig Length (bp)** | **Contig Number** | **Scaffold Length (bp)** | **Scaffold Number** |
| N90 | 13,467,797 | 31 | 13,467,797 | 31 |
| N80 | 21,117,751 | 26 | 21,117,751 | 26 |
| N70 | 24,617,574 | 22 | 24,617,574 | 22 |
| N60 | 27,825,520 | 18 | 27,825,520 | 18 |
| N50 | 30,574,287 | 15 | 30,574,287 | 15 |
| Total length | 973,197,345 | - | 973,197,345 | - |
| Number(>=100bp) | - | 88 | - | 88 |
| Number(>=2kb) | - | 88 | - | 88 |
| Max length | 36,793,432 | - | 36,793,432 | - |

Table S3. Statistical results of reads alignment for the *Sesamia inferens* genome.

| The version genome | Genome size (Mb) | Karyotype | Number of contigs | Number of scaffolds | Max scaffold length (Mb) | Contig N50 (kb) | Scaffold N50 (Mb) | BUSCO (%) | G + C (%) | Repeat (%) | Number of genes |
| --- | --- | --- | --- | --- | --- | --- | --- | --- | --- | --- | --- |
| The previously published^60^ | 865.04 | — | 1135 | 69 | 38 | 1231.9 | 28.6 | 96.1 | 38.62 | 53.14 | 18937 |
| This study | 973.18 | 30+Z | 88 | 88 | 36.79 | 30574.3 | 30.57 | 98.9 | 38.9 | 58.59 | 26628 |
